# Remarks on the Cooking and Preserving of Meat

**Published:** 1851-09

**Authors:** 


*Remarks on the Cooking and Preserving of Meat.* By Prof. Liebig.—The view that broth derives its nourishing properties essentially from the dissolved gelatin—an opinion which has frequently been discountenanced in practice—is shown by this investigation to be completely untenable. The gelatin imparts no taste to broth, and forms by far too insignificant a portion to allow of its nutritious properties being dependent upon it. Chopped beef, or veal, previously exhausted in the cold, when boiled for five hours, yielded to the broth, the former 0.5 per cent. and the latter 1.5 per cent. of soluble constituents, of which gelatin formed, at most, but one-half. On the contrary, this investigation confirms the view of Prout, that the peculiar constituents of broth exist ready formed in the flesh, and are by no means merely products of the process of ebullition. The residue of the chopped muscular flesh of different animals—as of the fox and ox—

after having been exhausted in the cold, cannot be distinguished the one from the other; all the peculiarities of the flesh, especially its flavour, depending entirely upon the soluble constituents which are found in the broth.

The researches of Liebig offer a simple and convenient method of preparing, in a few minutes, a broth of the highest nutritive properties. Finely-chopped lean beef is mixed with an equal weight of cold water, and left, if possible, to macerate for a short time, and the whole then slowly heated to ebullition. After gently boiling for some minutes, the clear broth separates from the coagulated albumen, and from the muscular fibre, which has now assumed a sinewy appearance. After straining, it requires only to be seasoned; and slightly colored with burnt onions, or with caramel. The coloring of the broth is nothing but a concession to the common prejudice, which cannot, however, be well dispensed with. By evaporation in a water-bath, or at a still lower temperature, the broth becomes spontaneously colored, and leaves behind a brown extract, possessing a delicate odour of roasted meat; this extract, when dissolved in about thirty parts of water, and flavored with salt, yields, at any moment, a most excellent broth. The advantage of extract of flesh for the nutrition of invalids, its use in hospitals, or in field service, as well as in domestic economy, is sufficiently obvious. We see, likewise, that bone-broth, broth-tablets, &c., being preparations essentially different from a true broth from flesh, cannot enter into competition with it as articles of food.

As an article of commerce, extract of flesh bears somewhat too high a price. It appears, however, to offer a new source of profit to the inhabitants of the different settlements in America and Australia, who might successfully prepare it from their cattle at a cheaper rate, and send it to the markets of our crowded populations.

As to the cooking of meat, it follows that to prepare, by boiling, a rich broth, and, at the same time, a savoury *bouilli*, is perfectly impossible. After preparing broth to the above directions, the meat which remains is perfectly unpalatable, tasteless, and tough, and as dissimilar as possible to the boiled beef of our tables. If, on the other hand, it be desirable to leave in the boiled meat the greatest amount of nutrition and flavour, it must be at once plunged into boiling water. If the temperature, after some minutes, be reduced to about 158° Fahr. by the addition of cold water, and the water maintained at that temperature until the meat is thoroughly cooked, all the conditions necessary for this purpose will have been fulfilled. If it be perfectly established that pure fleshy fibre —viewed independently of the juice—instead of being softened by boiling, is converted into a horny or sinewy mass, it is evident that this change is prevented by two different means in the ordinary mode of cooking meat: in the first place, by the temperature in the interior of the piece of meat never reaching the boiling heat; and, in the second place, by its being, nevertheless, sufficiently high to coagulate the albumen which surrounds, and, to a certain extent, protects the fibre. The temperature in the interior of the meat is not only sufficient to coagulate the albumen (132° Fahr.), but must attain even the point necessary for the coagulation of the colouring matter of the blood (from 149° to 158° Fahr.).

The investigation of Liebig exhibits the process of salting meat under a perfectly new aspect. The "brine," which meat and salt form when together, amounts to from one-third to one-half of the juice of the meat, and contains the chief constituents of concentrated broth. The brine presents an acid reaction, and, owing to the quantity of albumen present, coagulates when boiled; it contains moreover, phosphoric acid, lactic acid, a large amount of potassa, kreatinine, and, doubtlessly, also kreatine. There can be no doubt, therefore, that salting diminishes the nutricious properties of meat, by the amount of constituents which pass into the brine; hence the explanation of the well-known injurious effect on health produced by the continued consumption of salt meat.—*Liebig's Report*, vol. ii.

